# Association Between Leukocyte Mitochondrial DNA Copy Number and Non-alcoholic Fatty Liver Disease in a Chinese Population Is Mediated by 8-Oxo-2′-Deoxyguanosine

**DOI:** 10.3389/fmed.2020.00536

**Published:** 2020-09-10

**Authors:** Chifa Ma, Yiwen Liu, Shuli He, Jingbo Zeng, Pingping Li, Chunxiao Ma, Fan Ping, Huabing Zhang, Lingling Xu, Wei Li, Yuxiu Li

**Affiliations:** ^1^Key Laboratory of Endocrinology, Ministry of Health, Department of Endocrinology, Peking Union Medical College Hospital, Peking Union Medical College, Chinese Academy of Medical Sciences, Beijing, China; ^2^Department of Nutrition, Peking Union Medical College Hospital, Beijing, China; ^3^Department of Endocrinology, Fuxing Hospital, The Eighth Clinical Medical College, Capital Medical University, Beijing, China; ^4^State Key Laboratory of Bioactive Substance and Function of Natural Medicines, Institute of Materia Medica, Chinese Academy of Medical Sciences and Peking Union Medical College, Beijing, China; ^5^Diabetes Research Center of Chinese Academy of Medical Sciences, Beijing, China

**Keywords:** inflammation markers, leukocyte mitochondrial DNA copy number, non-alcoholic fatty liver disease, oxidative stress, 8-oxo-2′-deoxyguanosine

## Abstract

**Background:** Alterations in mitochondrial DNA are potentially associated with oxidative stress and may be involved in the pathogenesis of non-alcoholic fatty liver disease (NAFLD). However, the association between mitochondrial DNA copy number (mtDNAcn) and NAFLD was not consistent. In addition, the association between inflammation and NAFLD has not been established yet. The present study, based on a Chinese population of individuals with different glucose statuses, aimed to explore the association between leukocyte mtDNAcn, markers of oxidative stress, and inflammation and NAFLD.

**Methods:** A total of 318 participants from a diabetes project were included. NAFLD was diagnosed by ultrasonography. Leukocyte mtDNAcn was determined by PCR assay. The levels of the inflammation markers tumor necrosis factor α (TNF-α) and interleukin 6 (IL-6) and the oxidative stress markers glutathione reductase (GR), superoxide dismutase (SOD), and 8-oxo-2′-deoxyguanosine (8-oxo-dG) were also measured.

**Results:** Participants with NAFLD (*n* = 105) exhibited significantly higher leukocyte mtDNAcn, IL-6, and 8-oxo-dG (all *P* < 0.05). Pearson correlation analysis indicated mtDNAcn was negatively associated with age, uric acid, SOD, and TNF-α, but was positively associated with 8-oxo-dG (all *P* < 0.05). Univariate logistic regression analysis revealed that mtDNAcn was positively associated with NAFLD [odds ratio (OR) = 1.617, 95% confidence interval (CI) = 1.036–2.525; *P* = 0.034]. However, after adjustment for 8-oxo-dG, this association was no longer statistically significant (OR = 1.534, 95% CI = 0.979–2.403, *P* = 0.062). Moreover, the stress marker 8-oxo-dG was independently associated with NAFLD after adjustment for mtDNAcn, IL-6, glucose tolerance status, and other conventional NAFLD risk factors (OR = 1.707, 95% CI =1.142–2.550, *P* = 0.009). Mediation analysis indicated that 8-oxo-dG fully mediated the effect of mtDNAcn on the incidence of NAFLD (direct effect β = 0.5221, 95% CI = −0.0648 to 1.2504; indirect effect β = 0.0946, 95% CI = 0.0049–0.2463).

**Conclusions:** In a Chinese population, the association between leukocyte mtDNAcn and NAFLD was fully mediated by high levels of 8-oxo-dG. Thus, oxidative stress may be an important driver of NAFLD, and clinical interventions aimed at decreasing oxidative stress to improve NAFLD warrant further research.

## Introduction

Non-alcoholic fatty liver disease (NAFLD), which includes a number of hepatic pathologies ranging from liver steatosis to non-alcoholic steatohepatitis (NASH) and cirrhosis, is increasingly prevalent all over the world (1). In urban and rural areas of China, the morbidity of NAFLD has been reported to be > 20% (2). NAFLD can progress to more severe liver diseases and contribute to liver cancer with deleterious therapeutic effects and poor prognosis (3). Patients with NASH have higher mortality than the general population (4). Thus, there is an urgent need to find reliable markers to identify NAFLD at the early stages and to clarify its mechanisms, in order to facilitate clinical precautions and treatment.

Mitochondria play a significant role in providing energy and are the source of reactive oxygen species in the cell. Qualitative and quantitative alterations in mitochondrial DNA are therefore potentially associated with oxidative stress and many diseases (5–7). Oxidative stress is involved in the pathogenesis of NAFLD (8, 9). Levels of the oxidative stress marker 8-oxo-2′-deoxyguanosine (8-oxo-dG), a product of DNA oxidative damage, are increased in patients with NAFLD (10, 11). Our previous study, based on patients with type 2 diabetes mellitus, indicated that telomere length, one marker of cellular aging, could predict the incidence of NAFLD upon a 6-year follow-up (12). Because mitochondrial DNA copy number (mtDNAcn) has been closely associated with telomere length and oxidative stress (7, 13, 14), both of which are related to NAFLD, we hypothesized that mtDNAcn may also be associated with NAFLD. Nevertheless, studies on the association of NAFLD with liver mtDNAcn were contradictory. For instance, increased liver mtDNAcn has been reported in mice and patients with NAFLD (15, 16), whereas another study indicated that liver mtDNAcn was decreased in patients with NAFLD (17). A previous study has indicated that the mtDNAcn of leukocytes is well-related to that in the rat liver (18), but the studies on the association between leukocyte mtDNAcn and NAFLD are limited. An association between inflammation and NAFLD has not yet been established (19, 20). The present study, based on participants with different glucose tolerance statuses, aimed to explore the association between leukocyte mtDNAcn, markers of inflammation, and oxidative stress, as well as NAFLD.

## Materials and Methods

### Study Population and Clinical Measurement

**Figure 1:**
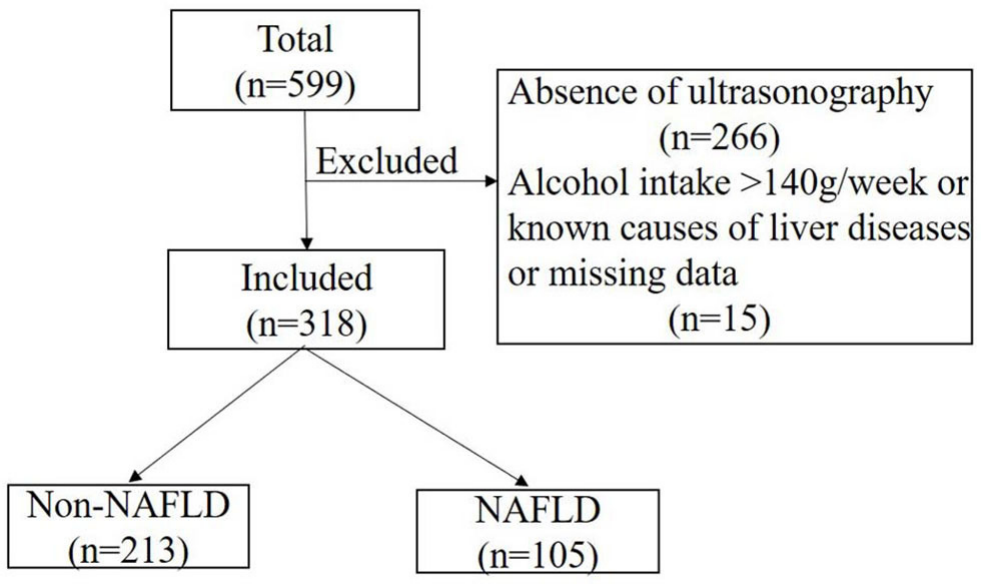
Flowchart of this study. NAFLD, non-alcoholic fatty liver disease.

A total of 599 participants were recruited from a diabetes project in a Beijing suburb between 2014 and 2015 (21), and 333 of them completed liver ultrasonography evaluation. Participants with an alcohol intake > 140 g/wk, known causes of liver disease, or missing data were excluded (*n* = 15). A final total of 318 participants were included (Figure 1). Medical history was obtained by questionnaire. Body mass index (BMI), blood pressure, and waist circumference (WC) were also collected using standard methods. Abnormal glucose tolerance (AGT), including diabetes and prediabetes as indicated by impaired fasting glucose and impaired glucose tolerance, was diagnosed according to the 1999 World Health Organization criteria after a 75-g oral glucose tolerance test.

### Biochemical Measurements

Fasting (> 10 h) blood samples were collected. Hemoglobin A_1c_ (HbA_1c_) was analyzed by high-performance liquid chromatography. Plasma glucose concentration was measured by a glucose oxidase assay. Plasma insulin was determined by chemiluminescent immunoassay. Homeostatic Model Assessment of Insulin Resistance (HOMA-IR) was used to evaluate insulin resistance (22). Plasma alanine transaminase (ALT), aspartate transaminase (AST), uric acid (UA), and lipids were measured by an automated analyzer. The levels of the inflammation markers tumor necrosis factor α (TNF-α) and interleukin 6 (IL-6) and the oxidative stress markers glutathione reductase (GR), superoxide dismutase (SOD), and 8-oxo-2′-deoxyguanosine were determined by kits according to the manufacturer's protocols (Cloud-Clone Corp, Houston, TX, USA).

### Measurement of mtDNAcn

Our previous studies described leukocyte mtDNAcn analysis in detail (21, 23). In brief, genomic DNA was extracted from leukocytes by QIAamp DNA Blood Midi Kits (Qiagen, Hilden, Germany). Purified DNA samples were diluted and quantified using a NanoDrop 1000 spectrophotometer (Thermo Fisher Scientific, Wilmington, DE, USA). The relative mtDNAcn was determined using real-time polymerase chain reaction and corrected by simultaneously measuring nuclear DNA.

### Liver Ultrasonography Evaluation

NAFLD was diagnosed by ultrasonography according to the guidelines for the diagnosis and management of NAFLD by the Chinese Society of Hepatology (2010).

Specifically, the presence of at least two out of three of the following ultrasonic characteristics was required: bright liver, liver echo greater than kidney, vascular blurring, and deep attenuation of ultrasound signal (24). Significant alcohol consumption or other known causes of liver disease were excluded before NAFLD diagnosis. The trained ultrasound physicians were blinded to the participants' medical history and biochemical measurement results.

### Statistical Analysis

Normally distributed quantitative variables were expressed as the mean ± standard deviation, whereas categorical variables were shown as percentages. Parameters that were not normally distributed were transformed or presented as the median (25–75th percentile). Comparison of variables between groups was performed using Student *t*-test or non-parametric Mann–Whitney *U*-test or χ^2^-test. Pearson correlation analysis was performed to determine the correlations between normally distributed variables and leukocyte mtDNAcn, whereas Spearman correlation analysis was used to test the associations between non-normally distributed parameters and leukocyte mtDNAcn. Univariate and multivariate logistic regression analyses were used to explore the association of mtDNAcn with NAFLD. Mediation model analysis was conducted to explore the potential mediating role of 8-oxo-dG in the association between mtDNAcn and NAFLD using PROCESS macro version 3.3 (25). Zero did not cross the 95% confidence interval (CI) estimated using bootstrap sampling (5,000 repetitions) representing the statistical significance of the mediating effect.

SPSS (version 22.0) was used to perform all statistical analyses. *P* < 0.05 (two-sided) indicated statistical significance.

## Results

### Clinical and Demographic Characteristics in the NAFLD and Non-NAFLD Groups

A total of 318 adults (36.8% male) aged 25–81 years were included, divided into a non-NAFLD group (*n* = 213) and a NAFLD group (*n* = 105). Table 1 presents the differences in characteristics between the groups. There were no statistical differences between groups regarding gender and age, whereas participants with NAFLD had higher BMI, WC, triglyceride (TG), low-density lipoprotein cholesterol (LDL-C), UA, HbA_1c_, HOMA-IR, ALT, and AST but had lower high-density lipoprotein cholesterol (HDL-C) compared to those with non-NAFLD (all *P* < 0.05). The prevalence of AGT was also higher in the NAFLD group (70.5 vs. 54.5%, *P* = 0.006).

**Table 1:** Clinical and demographic characteristics in the non-alcoholic fatty liver disease (NAFLD) and non-NAFLD groups.

	**Non-NAFLD**	**NAFLD**	***P***
n (%)	213 (66.98)	105 (33.02)	
Gender, n (%)			0.515
Female, n (%)	132 (62)	69 (65.7)	
Male, n (%)	81 (38.0)	36 (34.3)	
Age, years	53.58 ± 11.18	52.51 ± 9.09	0.363
BMI, kg/m^2^	24.95 ± 3.11	28.56 ± 3.87	<0.001
WC, cm	85.42 ± 8.82	92.13 ± 9.39	<0.001
SBP, mmHg	128.35 ± 19.55	129.39 ± 17.23	0.642
DBP, mmHg	75.22 ± 9.87	76.62 ± 9.81	0.235
TC, mmol/L	5.44 ± 1.06	5.64 ± 1.13	0.109
LnTG, mmol/L	0.27 ± 0.53	0.67 ± 0.55	<0.001
HDL-C, mmol/L	1.33 ± 0.38	1.20 ± 0.22	0.002
LDL-C, mmol/L	2.80 ± 0.73	3.04 ± 0.71	0.005
UA, μmol/L	280.29 ± 74.64	307.97 ± 86.21	0.003
HbA_1c_, %	5.5 (5.2–5.9)	5.7 (5.5–6.3)	<0.001
LnHOMA-IR	0.84 ± 0.63	1.34 ± 0.56	<0.001
ALT, U/L	21.2 (16.8–28.18)	31.25 (21.93–42.95)	<0.001
AST, U/L	21.80 (18.7–25.18)	23.7 (20.55–31.8)	<0.001
Glucose tolerance status			0.006
NGT, n (%)	97 (45.5)	31 (29.5)	
AGT, n (%)	116 (54.5)	74 (70.5)	

*Data are presented as mean ± standard deviation, number (percentage), or median (25–75th percentile). BMI, body mass index; WC, waist circumference; SBP, systolic blood pressure; DBP, diastolic blood pressure; TC, total cholesterol; TG, triglycerides; HDL-C, high-density lipoprotein cholesterol; LDL-C, low-density lipoprotein cholesterol; UA, uric acid; HbA_1c_, hemoglobin A_1c_; HOMA-IR, Homeostatic Model Assessment of Insulin Resistance; ALT, alanine aminotransferase; AST, aspartate aminotransferase; NGT, normal glucose tolerance; AGT, abnormal glucose tolerance*.

**Figure 2:**
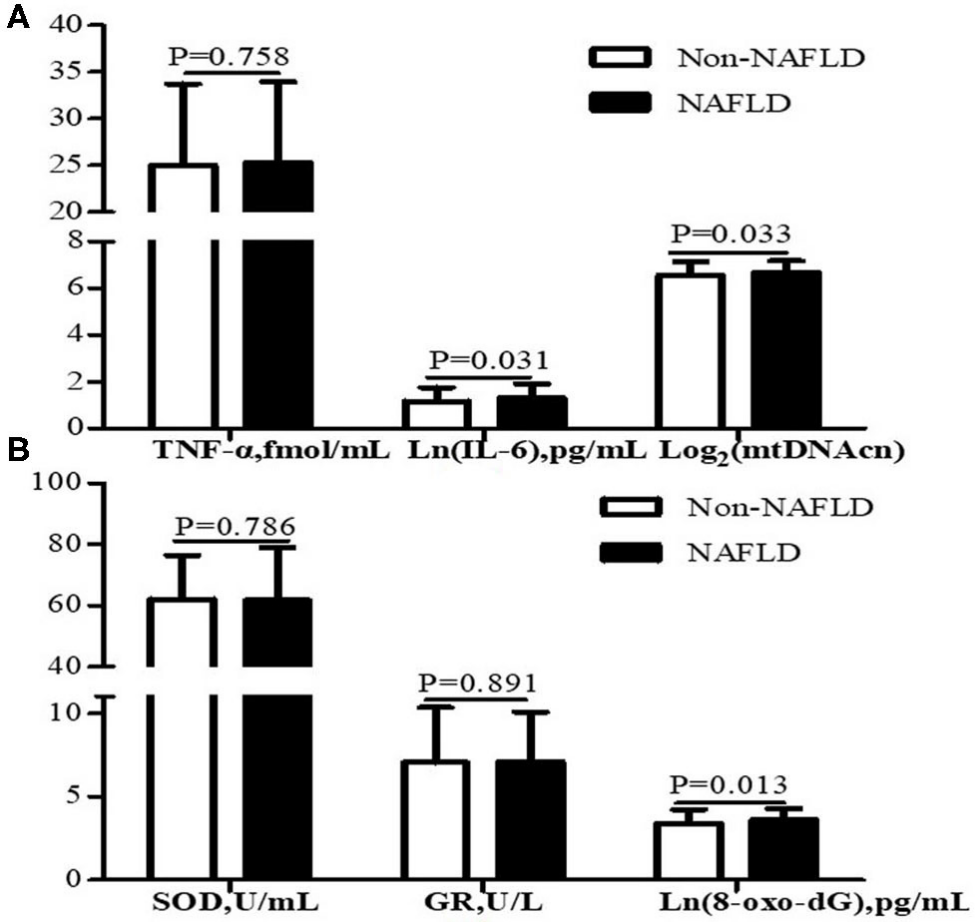
Leukocyte mtDNAcn and inflammation markers **(A)** and oxidative stress markers **(B)** of participants with NAFLD (*n* = 105) and without NAFLD (*n* = 213). Data are presented as mean ± standard deviation. Participants with NAFLD exhibited higher IL-6, mtDNAcn, and 8-oxo-dG than those without NAFLD (*P* < 0.05). NAFLD, non-alcoholic fatty liver disease; mtDNAcn, mitochondrial DNA copy number; SOD, superoxide dismutase; GR, glutathione reductase; 8-oxo-dG, 8-oxo-2′-deoxyguanosine; TNF-α, tumor necrosis factor α; IL-6, interleukin 6.

As shown in Figure 2, participants with NAFLD exhibited higher mtDNAcn (6.70 ± 0.48 vs. 6.55 ± 0.59, *P* = 0.033), 8-oxo-dG (3.62 ± 0.68 vs. 3.40 ± 0.84 pg/mL, *P* = 0.013), and IL-6 (1.31 ± 0.60 vs. 1.16 ± 0.60 pg/mL, *P* = 0.031) than those without NAFLD, whereas we failed to observe a statistical difference in SOD (*P* = 0.786), GR (*P* = 0.891), or TNF-α (*P* = 0.758) between the two groups.

### Correlation of Leukocyte mtDNAcn With Potential Risk Factors of NAFLD

The correlations between leukocyte mtDNAcn and potential risk factors of NAFLD are recorded in Table 2. Correlation analysis indicated that age (*r* = −0.123, *P* = 0.029), UA(*r* = −0.136, *P* = 0.015), TNF-α (*r* = −0.195, *P* < 0.001), and SOD (*r* = −0.135, *P* = 0.016) were inversely associated with mtDNAcn, whereas 8-oxo-dG was the only factor that was positively associated with mtDNAcn (*r* = 0.125, *P* = 0.026).

**Table 2:** Correlation of leukocyte mitochondrial DNA copy number with potential risk factors of non-alcoholic fatty liver disease.

	**Log**_****2****_**(mtDNAcn)**	
	***r***	***P***
Age^†^	−0.123	0.029
BMI^†^	0.071	0.204
WC^†^	0.001	0.992
SBP^†^	0.025	0.654
DBP^†^	0.005	0.924
TC^†^	0.012	0.826
LnTG^†^	0.059	0.293
HDL-C^†^	−0.091	0.107
LDL-C^†^	0.028	0.618
UA^†^	−0.136	0.015
HbA1c^‡^	−0.068	0.225
LnHOMA-IR^†^	0.036	0.522
ALT^‡^	−0.045	0.423
AST^‡^	−0.039	0.492
ln(IL-6)^†^	−0.105	0.061
TNF-α^†^	−0.195	<0.001
Ln(8-oxo-dG)^†^	0.125	0.026
SOD^†^	−0.135	0.016
GR^†^	0.003	0.953

*^†^Non-normally distributed parameters. ^‡^Normally distributed parameters*.

*Pearson correlation analysis was performed to determine the correlations between normally distributed variables and mtDNAcn, whereas Spearman correlation analysis was used to test the associations between non-normally distributed parameters and mtDNAcn*.

*mtDNAcn, mitochondrial DNA copy number; BMI, body mass index; WC, waist circumference; SBP, systolic blood pressure; DBP, diastolic blood pressure; TC, total cholesterol; TG, triglycerides; HDL-C, high-density lipoprotein cholesterol; LDL-C, low-density lipoprotein cholesterol; UA, uric acid; HbA_1c_, hemoglobin A_1c_; HOMA-IR, Homeostatic Model Assessment of Insulin Resistance; ALT, alanine aminotransferase; AST, aspartate aminotransferase; NGT, normal glucose tolerance; AGT, abnormal glucose tolerance; IL-6, interleukin 6; TNF-α, tumor necrosis factor; 8-oxo-dG, 8-oxo-2′-deoxyguanosine; SOD, superoxide dismutase; GR, glutathione reductase*.

### Association of Leukocyte mtDNAcn With NAFLD

Logistic regression models of leukocyte mtDNAcn with NAFLD are presented in Table 3. Univariate logistic regression analysis showed that mtDNAcn [odds ratio (OR) = 1.617, 95% CI = 1.036–2.525; *P* = 0.034] was positively associated with NAFLD (Model1). However, following further adjustment for 8-oxo-dG, the association between mtDNAcn and NAFLD was not statistically significant (OR = 1.534, 95% CI = 0.979–2.403, *P* = 0.062, Model 2), suggesting the association between mtDNAcn and NAFLD might depend on 8-oxo-dG to some extent. In addition, 8-oxo-dG (OR = 1.707, 95% CI = 1.142–2.550, *P* = 0.009) was significantly associated with NAFLD, independent of age, gender, BMI, WC, UA, glucose tolerance status, HOMA-IR, blood pressure, lipids, ALT, AST, mtDNAcn, and IL-6 (Model 3).

**Table 3:** Logistic regression analysis for association of leukocyte mitochondrial DNA copy number with non-alcoholic fatty liver disease.

**Variables**	**OR (95%CI)**	***P***
**Model 1**		
Log_2_ (mtDNAcn)	1.617 (1.036, 2.525)	0.034
**Model 2**		
Log_2_ (mtDNAcn)	1.534 (0.979, 2.403)	0.062
Ln (8-oxo-dG)	1.399 (1.020, 1.919)	0.037
**Model 3**		
Log_2_ (mtDNAcn)	1.686 (0.933, 3.044)	0.083
Ln (8-oxo-dG)	1.707 (1.142, 2.550)	0.009
Gender		
Female	Reference	
Male	0.977 (0.468, 2.043)	0.951
Age	0.988 (0.957, 1.020)	0.451
BMI	1.222 (1.093, 1.367)	<0.001
WC	1.022 (0.981, 1.064)	0.299
HDL-C	0.537 (0.116, 2.490)	0.427
LDL-C	1.302 (0.824, 2.057)	0.258
LnTG	1.905 (0.943, 3.849)	0.073
UA	0.999 (0.995, 1.003)	0.650
Glucose status		
Normal glucose tolerance	Reference	
Pre-DM + DM^†^	1.142 (0.589, 2.214)	0.695
LnHOMA-IR	1.743 (0.995, 3.051)	0.052
SBP	1.001 (0.984, 1.018)	0.927
DBP	0.997 (0.966, 1.029)	0.835
ALT	1.007 (0.985, 1.030)	0.527
AST	1.060 (0.996, 1.127)	0.065
Ln (IL-6)	1.670 (0.984, 2.835)	0.057

*OR, odds ratio; CI, confidential interval; mtDNAcn, mitochondrial DNA copy number; 8-oxo-dG, 8-oxo-2′-deoxyguanosine; BMI, body mass index; WC, waist circumference; HDL-C, high-density lipoprotein cholesterol; LDL-C, low-density lipoprotein cholesterol; TG, triglyceride; UA, uric acid; DM, diabetes mellitus; HOMA-IR, Homeostatic Model Assessment of Insulin Resistance; SBP, systolic blood pressure; DBP, diastolic blood pressure; ALT, alanine aminotransferase; AST, aspartate aminotransferase; IL-6, interleukin 6*.

*^†^Pre-DM (including impaired fasting glucose and impaired glucose tolerance) and DM were diagnosed according to the 1999 World Health Organization criteria*.

**Figure 3:**
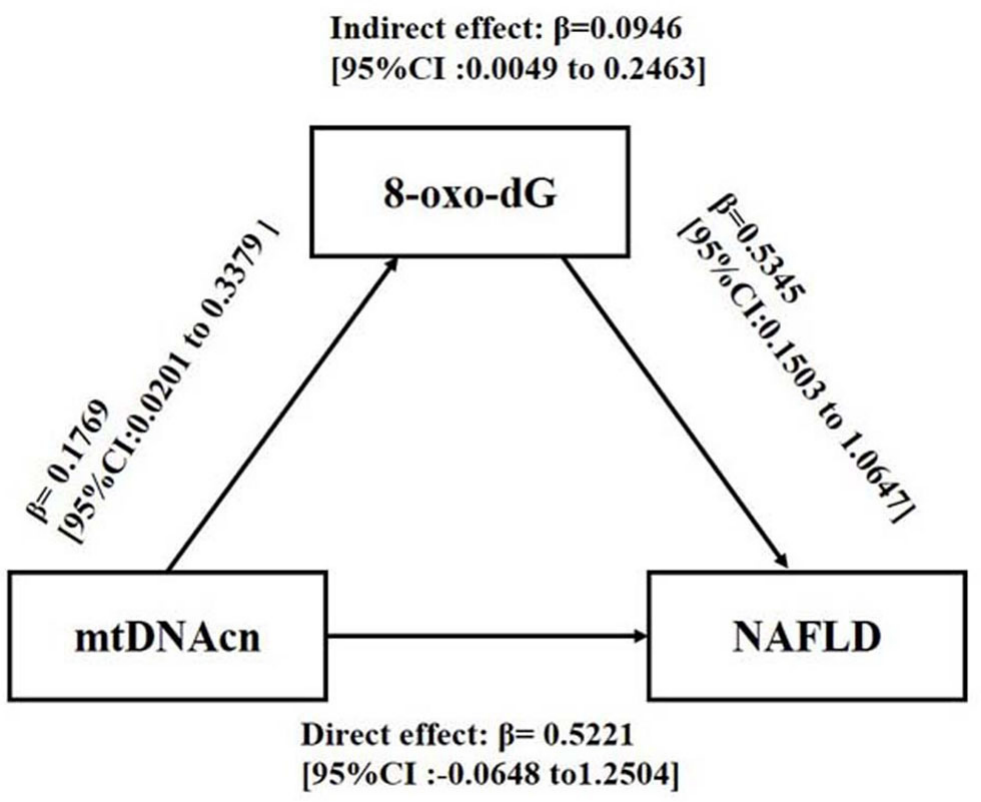
Mediation effect of 8-oxo-2′-deoxyguanosine (8-oxo-dG) on the association between leukocyte mitochondrial DNA copy number (mtDNAcn) and non-alcoholic fatty liver disease (NAFLD). Models were adjusted by gender, age, body mass index, waist circumference, high-density lipoprotein cholesterol, low-density lipoprotein cholesterol, triglyceride, uric acid, glucose tolerance status, Homeostatic Model Assessment of Insulin Resistance, systolic blood pressure, diastolic blood pressure, alanine aminotransferase, aspartate aminotransferase, and interleukin 6. The effect was presented as β [95% confidence interval (CI)]. Zero did not cross the 95% CI representing statistical significance.

To explore whether 8-oxo-dG mediated the effect of mtDNAcn on NAFLD, a mediation analysis was conducted after adjusting for gender, age, BMI, WC, HDL-C, LDL-C, TG, UA, glucose tolerance status, HOMA-IR, blood pressure, ALT, AST, and IL-6. As shown in Figure 3, mtDNAcn had no direct effect on NAFLD (direct effect β = 0.5221, 95% CI = −0.0648 to 1.2504), whereas 8-oxo-dG fully mediated the effect of mtDNAcn on the incidence of NAFLD (indirect effect β = 0.0946, 95% CI = 0.0049–0.2463).

## Discussion

The present study, based on a Chinese population with different glucose tolerance statuses, found that participants with NAFLD had higher leukocyte mtDNAcn, IL-6, and 8-oxo-dG levels, as well as higher levels of conventional NAFLD risk factors. Mediation analysis indicated that 8-oxo-dG fully mediated the effect of mtDNAcn on the incidence of NAFLD, suggesting the important role oxidative stress may play in the pathogenesis of NAFLD. Although glucose tolerance status was adjusted in this analysis, participants with NAFLD were more likely to suffer from AGT (70.5 vs. 54.5%, *P* = 0.006), and the level of mtDNAcn and oxidative stress markers may be affected by different glucose tolerances (21). In order to eliminate the interference influence of different glucose tolerance, participants were divided into normal glucose tolerance (NGT) and AGT groups. However, there were no significant differences in mtDNAcn, inflammation, or oxidative stress markers between participants with NGT and AGT (all *P* > 0.05, Supplementary Figure 1), which suggested glucose tolerance was not a significant confounding factor that affected the association between NAFLD and mtDNAcn in the present study.

In the present study, participants with NAFLD had higher mtDNAcn, in agreement with previous studies (16, 26). Malik et al. (16) found elevated liver mtDNAcn in high-fat diet–induced mice. A clinical study also indicated that non-diabetic hemodialysis patients with NAFLD had significantly higher leukocyte mtDNAcn than those without NAFLD (26). A study by Sookoian et al. (17), however, suggested that liver mtDNAcn was decreased in patients with NAFLD. A clinical study showed that alterations in leukocyte mtDNAcn were associated with the clinicopathological severity of NAFLD (27), which could partly explain the discrepancy among these studies. However, when studying the association between mtDNAcn and NAFLD, the effect of potential confusing factors such as inflammation and oxidative stress markers was seldom taken into account simultaneously by most researchers. We addressed this issue in the present study. Besides leukocyte mtDNAcn, NAFLD patients in the present study also had higher levels of 8-oxo-dG. A previous study has indicated that patients and mice with NASH exhibited increased mtDNA and oxidative stress (28). Furthermore, we found that 8-oxo-dG fully mediated the effect of mtDNAcn on the incidence of NAFLD, suggesting that oxidative stress plays a vital role in the pathogenesis of NAFLD. Several studies have also reported that oxidative stress may play a significant role in the production of NAFLD (8, 9). We conducted an in-depth analysis of the complicated relationship between mtDNAcn, oxidative stress, and NAFLD. The mtDNAcn is an indicator of the number of mitochondria, which is determined by their rates of biogenesis and degradation. Mild oxidative stress is one of the abnormal signals that can contribute to mitochondrial biogenesis and the upregulation of mtDNAcn (29). On the other hand, under excess oxidative stress conditions, the cell's ability to remove damaged mitochondria decreases, contributing to the accumulation of damaged mitochondria and mtDNA, as well as increased mtDNAcn, resulting in excess free radicals and enhanced oxidative stress (7). For instance, in a high-fat diet–induced liver steatosis rat model, increased levels of mtDNA and non-functional and damaged mtDNA were observed (16), which may have promoted mitochondrial dysfunction and oxidative stress and exacerbated NAFLD. A clinical study demonstrated that obese humans without NASH exhibited higher maximal respiration rates in isolated mitochondria from liver as compensation, whereas obese patients with NASH lost this compensation, despite higher mitochondrial numbers, suggesting the adaptation of the liver is lost as the disease progresses (30). In our present study, although some participants had higher mtDNAcn, this was positively associated with 8-oxo-dG rather than with antioxidants, suggesting that this increased mtDNA may promote oxidative stress and consequently cause NAFLD. Based on our results and conclusions from the literature (7, 16, 27, 29, 30), we hypothesize the following mechanism: (1) At an early stage, some individuals exhibit higher than normal levels of mitochondria and mtDNAcn due to mild oxidative stress. (2) Persistent oxidative stress goes beyond the mitochondrial ability to compensate, and damaged mitochondria and mtDNA accumulate. This response, in turn, promotes further oxidative stress and consequently the occurrence and development of NAFLD despite higher mtDNAcn. (3) Long-time oxidative stress could eventually lead to depletion of mtDNAcn. This hypothesis contributes to explaining the different results of studies into the level of mtDNAcn in NAFLD and highlighting the vital position of oxidative stress in the pathogenesis of NAFLD. However, longitudinal, as well as mechanistic, studies are needed.

IL-6 is one kind of proinflammatory cytokine and is closely associated with many metabolic disorders. Although the role of IL-6 in the development of NAFLD has not yet been established, a previous study indicated that participants with NASH exhibited elevated plasma and hepatic IL-6 levels (31). Consistent with this study, the present study also indicated that participants with NAFLD had high levels of plasma IL-6, whereas multivariate logistic regression analysis indicated that IL-6 was not an independent risk factor for NAFLD. Moreover, abundant evidence indicated that the role of IL-6 in the progression of NAFLD remained confusing. On the one hand, it was shown that IL-6 enhanced fatty acid synthesis in human primary hepatocytes (20). On the other hand, IL-6–related signaling has been reported to play a protective role in improving NAFLD in mice (19). The role of IL-6 in the progression of NAFLD needs further exploration. TNF-α is another proinflammatory cytokine. It has been reported that participants with NASH had higher TNF-α levels than controls, but there was no significant difference between participants with NASH and liver steatosis (32). The present study also found that participants with NAFLD had slightly higher TNF-α, but the trend did not reach statistical significance. Differences in severity of NAFLD and different study designs may contribute to these discrepancies. Therefore, further studies are needed to explore the association between inflammation markers and NAFLD.

The present study had some advantages. First, we revealed the possible mechanism of changes in leukocyte mtDNAcn levels in participants with NAFLD, using different statistical methods involving the analysis of multiple potential confusing factors such as oxidative stress and inflammation markers. Our findings improve the understanding of the association between mtDNAcn, inflammation and oxidative stress markers, and NAFLD. Second, participants with different glucose tolerances made the results more representative to some extent. However, several limitations should not be ignored. First, although ultrasonography is a common and convenient method to detect NAFLD, more accurate methods were not applied to diagnose NAFLD and determine the severity of NAFLD. Second, our study sample was relatively small. A larger cohort and more longitudinal studies are needed to clarify the details of the mechanism of NAFLD.

## Conclusions

In a Chinese population of individuals with different glucose tolerance statuses, we found that the association between leukocyte mtDNAcn and NAFLD was fully mediated by higher levels of 8-oxo-dG. Thus, oxidative stress may be an important driver of NAFLD, and clinical interventions targeting decreasing oxidative stress to improve NAFLD warrant further research. Further longitudinal clinical studies focusing on the pathological mechanisms underlying NAFLD are needed.

## Data Availability Statement

The datasets generated for this study are available on request to the corresponding author.

## Ethics Statement

The studies involving human participants were reviewed and approved by the Ethics Committee of Peking Union Medical College Hospital. The patients/participants provided their written informed consent to participate in this study.

## Author Contributions

ChiM: conducted the research, performed the statistical analysis, and wrote the draft. YLiu, SH, JZ, PL, ChuM, FP, HZ, LX, and WL: conducted the research and collected the data. YLi: designed the study and revised the manuscript. All authors read and approved the submitted version.

## Conflict of Interest

The authors declare that the research was conducted in the absence of any commercial or financial relationships that could be construed as a potential conflict of interest.

## Abbreviations

mtDNAcn: mitochondrial DNA copy number
NAFLD: non-alcoholic fatty liver disease
TNF-α: tumor necrosis factor α
IL-6: interleukin 6
GR: glutathione reductase
SOD: superoxide dismutase
8-oxo-dG: 8-oxo-2′-deoxyguanosine
NASH: non-alcoholic steatohepatitis
WC: waist circumference
AGT: abnormal glucose tolerance
NGT: normal glucose tolerance
HbA_1c_: hemoglobin A_1c_
HOMA-IR: Homeostatic Model Assessment of Insulin Resistance
ALT: alanine transaminase
AST: aspartate transaminase
UA: uric acid
HDL-C: high-density lipoprotein cholesterol
LDL-C: low-density lipoprotein cholesterol
TC: cholesterol
TG: triglyceride.
