# Supplementary material for: Association Between Leukocyte Mitochondrial DNA Copy Number and Non-alcoholic Fatty Liver Disease in a Chinese Population Is Mediated by 8-Oxo-2′-Deoxyguanosine
Source: Front Med (Lausanne). 2020 Sep 10;7:536. doi: 10.3389/fmed.2020.00536 (PMC7511508; doi:10.3389/fmed.2020.00536)
Supplement: Supplementary file 1 [file Presentation_1.PPTX]

## Slide 1
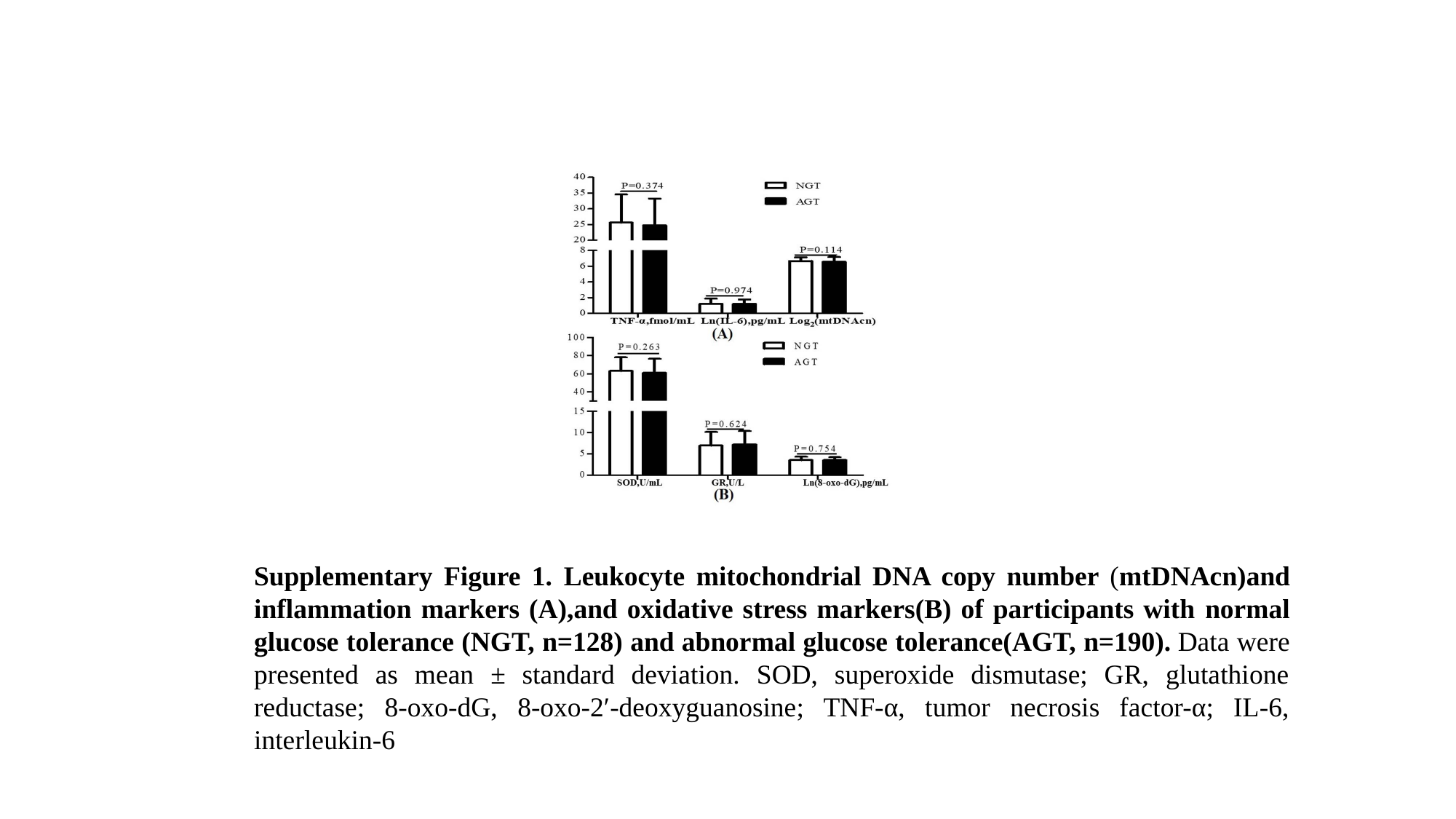

Supplementary Figure 1. Leukocyte mitochondrial DNA copy number (mtDNAcn)and inflammation markers (A),and oxidative stress markers(B) of participants with normal glucose tolerance (NGT, n=128) and abnormal glucose tolerance(AGT, n=190). Data were presented as mean ± standard deviation. SOD, superoxide dismutase; GR, glutathione reductase; 8-oxo-dG, 8-oxo-2′-deoxyguanosine; TNF-α, tumor necrosis factor-α; IL-6, interleukin-6
